# DataVersify: A Framework for Data Literacy Instruction Featuring Scientist Stories

**DOI:** 10.1093/iob/obag045

**Published:** 2026-08-06

**Authors:** E H Schultheis, R S Bellard, R A Costello, C D K Graham, M K Kjelvik, A Thadi, C J Ballen

**Affiliations:** Kellogg Biological Station, Michigan State University, Hickory Corners, MI 49060, USA; Department of Biological Sciences, Auburn University, Auburn, AL 36849, USA; Department of Biological Sciences, University at Buffalo, Buffalo, NY 14260, USA; Department of Biological Sciences, University at Buffalo, Buffalo, NY 14260, USA; Biological Sciences Department, Oakland University, Rochester, MI 48309, USA; Kellogg Biological Station, Michigan State University, Hickory Corners, MI 49060, USA; Department of Biological Sciences, Auburn University, Auburn, AL 36849, USA; Department of Biological Sciences, University at Buffalo, Buffalo, NY 14260, USA; Department of Biological Sciences, Auburn University, Auburn, AL 36849, USA

## Abstract

Developing data literacy is a core goal of biology education, yet many students struggle to engage with scientific research and data. DataVersify is a resource designed to support data literacy while simultaneously humanizing science. These activities integrate two established programs, Data Nuggets and Project Biodiversify, to engage students in the work of scientists and introduce them to the people behind the research. Within each activity, students encounter a scientist profile, read about a study, explore and visualize a dataset, construct evidence-based explanations, and ask questions of their own. Here, we synthesize over a decade of resource development and large-scale, coordinated research efforts to share the effective features of DataVersify activities. We found that pairing data literacy activities with humanizing details about a scientist’s life in and out of science increases students’ ability to relate to scientists and engage with course materials. We present six evidence-based recommendations for using DataVersify, including emphasize authenticity, pair data with scientist stories, and highlight a variety of contemporary scientist role models. Together, our work demonstrates how integrating authentic data experiences with scientist stories can support student outcomes in data literacy and biology education.

## Introduction

### The challenge—teaching data literacy in biology

Data literacy involves the ability to apply the appropriate quantitative and analytical tools necessary to analyze, interpret, synthesize, and communicate results from data (Mayes et al. 2014; Kjelvik and Schultheis 2019). Developing these abilities prepares life science students for advanced coursework and careers in biology, a field that increasingly depends on quantitative competencies (Brewer and Smith 2011; Gibson and Mourad 2018). Despite the importance of data literacy, students with low incoming math skills may perceive in-class quantitative problems as less useful than other in-class activities and report less enjoyment of math (Andrews and Aikens 2018). Students may also believe that quantitative problems in biology are unrelated to their everyday lives (Andrews and Aikens 2018) and those who conduct the work are out of reach and unrelatable (Wood et al. 2020). These perceptions can be reinforced by how science is represented in instructional materials (Damschen et al. 2005). For example, a recent analysis of ecology textbooks showed 76% of featured scientists were white men, and only 4% of textbook images contained any people at all, portraying ecology as a discipline largely detached from human experience (Richards et al. 2024). Thus, teaching materials that integrate science with quantitative data (Barsoum et al. 2013), along with examples highlighting the humanizing aspects of science with relatable scientist role models (Schultheis et al. 2024), may help students connect to the field of science, technology, engineering, and mathematics (STEM) and enhance engagement in biology and other science fields.

### A solution—DataVersify

**Fig. 1:**
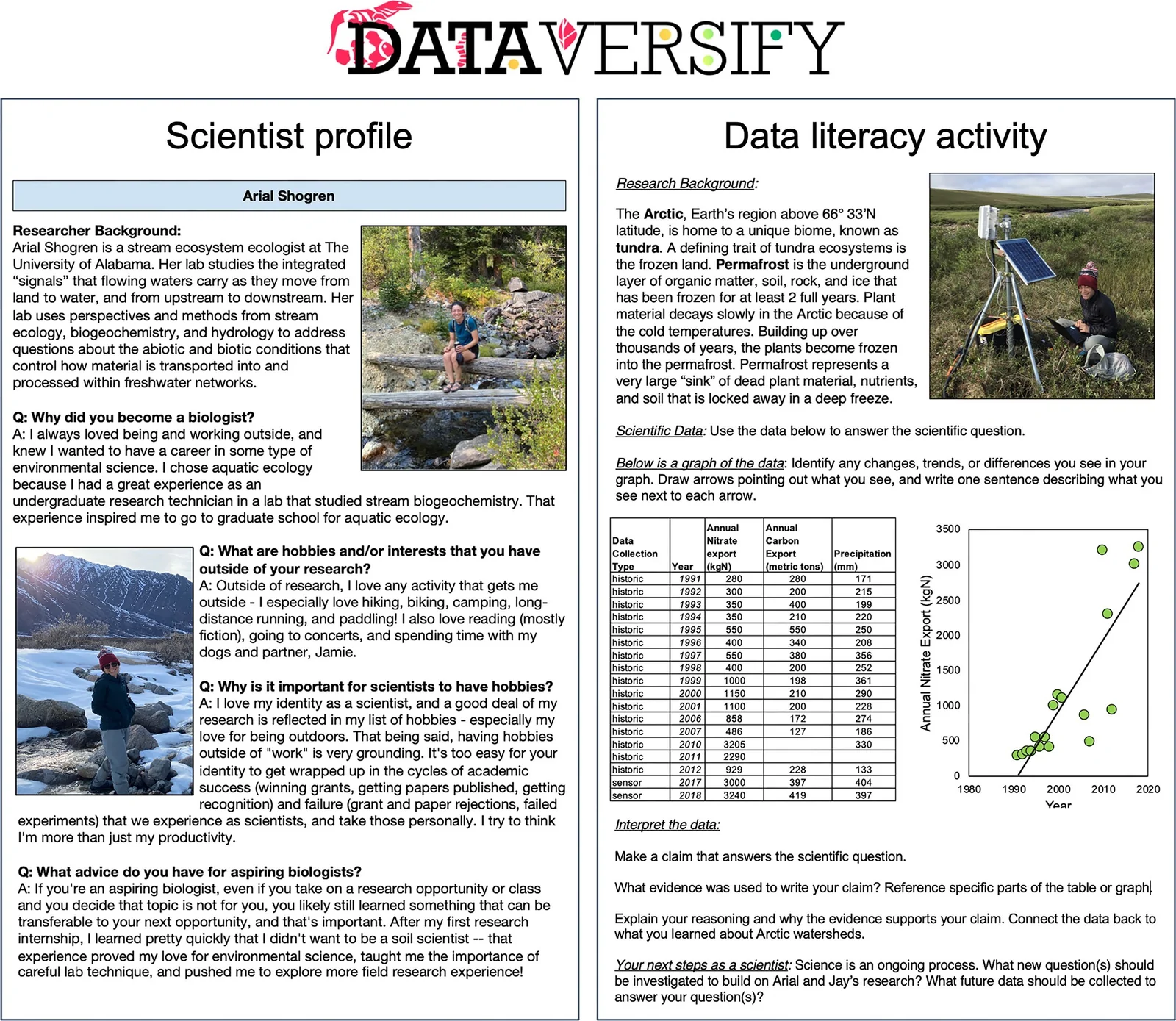
Anatomy of a DataVersify activity, which consists of a Scientist profile (left) paired with a Data Nuggets data literacy activity (right). All activities can be viewed and downloaded for free at https://datanuggets.org/dataversify.

**Fig. 2:**
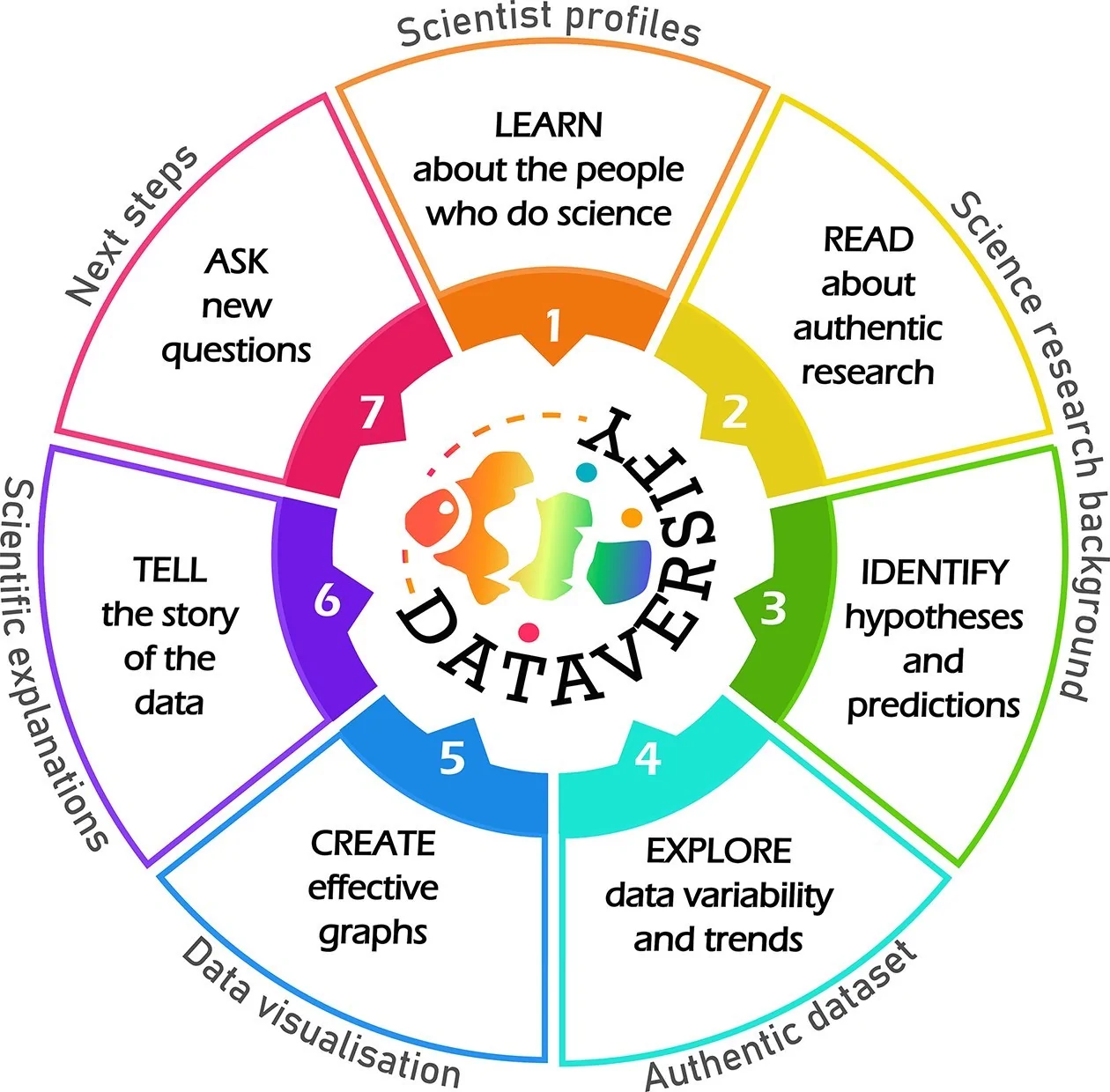
DataVersify learning objectives paired with template sections.

DataVersify materials support data literacy instruction by providing K-12 and undergraduate educators with activities featuring authentic research and data generated by contemporary scientists. When integrated into typical classroom instruction, these activities engage students in the practices of science, including reading scientific texts, asking questions, exploring data variability and trends, creating graphs, and constructing explanations. In addition to learning disciplinary biology content, students are introduced to the people behind the science, learning about the life and interests of contemporary scientists (Figs. 1 and 2).

DataVersify materials emerged from a unique collaboration between two established education programs: Data Nuggets and Project Biodiversify. Data Nuggets, founded in 2011 by scientists and educators, are targeted data literacy activities that share authentic research and datasets with students (Schultheis and Kjelvik 2015). Featuring a scientist and their work, Data Nuggets engage students in the practices of science as they answer a scientific question (Schultheis and Kjelvik 2015,  2020). They are written in collaboration with the scientists behind the research, ensuring they capture the unique story, interests, and voice of each individual. In high school biology classrooms, Data Nuggets have been shown to increase student interest in STEM careers, confidence in their ability to complete data literacy tasks, and ability to use data as evidence to support a claim (Schultheis et al. 2022). Project Biodiversify aims to “humanize biology as a field” by increasing the visibility of counter-stereotypical examples of scientist role models in introductory biology courses (Zemenick et al. 2022). They consist of scientist profiles, written in interview format, that highlight the personal lives and identities of featured scientists. Combined, these two programs became unified under the name “DataVersify”, promoting data literacy and exposing students to the lives and research of practicing scientists (Fig. 1).

DataVersify materials offer an opportunity to examine the influence of scientist role models—individuals whom students may choose to emulate. Role models in science help shape students’ perceptions of who can do science (Gladstone and Cimpian 2021) and provide well-documented benefits for students (Schinske et al. 2016; Gladstone and Cimpian 2021; Schultheis et al. 2024). Using DataVersify as a model system for education research, our team has built on the current understanding of scientist role models to help identify the particular elements of scientist stories that are most impactful for students, and how the match between student identity and the featured scientists impacts student outcomes. Research findings informed the iterative development and refinement of DataVersify activities as we incorporated best practices for introducing scientist role models in biology education.

In the following sections, we summarize what we have learned through resource development and research. We begin by describing the anatomy of a DataVersify activity, highlighting its core components, theoretical foundation, learning objectives, the process of data exploration and discovery for students, and tasks designed to promote quantitative competencies and critical thinking. Next, we share our research findings to date and offer six evidence-based suggestions to maximize the impact of DataVersify activities as instructors plan to integrate them in their biology courses. Finally, we conclude by looking forward into the future of this program, considering both curricular expansion and research directions.

## Anatomy of a DataVersify activity

DataVersify activities are built on a shared template (Fig. 1), the design of which draws on conversations with experienced educators and multiple theoretical frameworks, including metarepresentational competence, situated learning theory, self-determination theory, and other motivational theories, summarized below. These materials operationalize several learning objectives that emerge from educational reform standards, including *Vision and Change* (Brewer and Smith 2011; Clemmons et al. 2020), *AP College Readiness Standards* (College Board 2013), *Common Core State Standards* (Common Core 2014), and *Next Generation Science Standards* Practices (NGSS Lead States 2013). The DataVersify template consists of six main components: (1) scientist profile, (2) science research background, (3) authentic dataset, (4) data visualization, (5) constructing scientific explanations, and (6) next steps (Fig. 2).

Over the course of the DataVersify activity students shift from passive observers of science (i.e., reading about the life and work of a scientist) to active practitioners of science, generating their own questions and considering different approaches to conduct research. While all activities share a consistent structure, individual activities include learning objectives tailored to the scientist’s story, the particulars of the research study, the science content covered, and the type of dataset collected by the scientist.

DataVersify activities are designed to take about one to two class periods, and to be used repeatedly across academic terms and grade levels. They can be used as stand-alone activities, or integrated into a topic being taught in class. DataVersify activities can be assigned ahead of class time for flipped instruction, or as a homework assignment. Because activities follow a consistent structure and flow, they are easy for students to use and for instructors to implement. DataVersify activities are available for free, and are searchable by science content, grade level alignment, and data literacy skills (https://datanuggets.org/dataversify/). The activities range from those appropriate for 4th grade or above (Level 1) to those designed for upper-level high school or undergraduate classrooms (Level 4).

*Scientist profiles.* Each DataVersify activity starts with a scientist profile, in which students learn about the people who do science (Schultheis et al. 2024). While many of the studies presented are collaborative, one scientist is highlighted in each profile, though DataVersify activities may have several associated profiles from which an instructor or students may choose. Profiles are in question-and-answer, interview-style format that invite scientists to share their stories from both within and beyond their careers. This format encourages scientists to highlight personal aspects including their hobbies, pathways into their area of research, obstacles they have faced and overcome in their careers, and photos of their lives at work and play (Schultheis et al. 2024). Example prompts include:

1. Why did you become a scientist?
2. What is your favorite part about your job?
3. Please tell us about an obstacle you faced related to/during your STEM career.
4. What advice do you have for aspiring scientists?
5. Do you feel that any dimension of your identity is invisible or under-represented/marginalized in STEM?

From a theoretical perspective, scientist role models can affect key processes that support students in STEM and alter students’ motivation (reviewed in Gladstone and Cimpian 2021). For example, students who observe role models succeeding at a task have higher self-efficacy (Social Cognitive Theory; Bandura 2001; Ewen 2003). Exposure to successful role models can also increase the expectancy (“can I do this?”) and value (“do I care about this?”) that students assign to a task or goal (Situated Expectancy-Value Theory; Eccles and Wigfield 2020). Further, scientist role models can provide students with examples of ways to overcome various challenges they may face, increasing their motivation by suggesting strategies that help them gain autonomy, create relationships, and demonstrate competence (Self Determination Theory; Deci and Ryan 1985; Ryan and Deci 2000).

*Science research background.* Following the scientist profile, students engage in a Data Nuggets data literacy activity, which guides them through the full process of science as experienced by the researcher investigating a scientific question (Schultheis and Kjelvik 2015). The investigation begins with a research background in which students learn about authentic research and identify hypotheses and predictions. Narrative elements within this section, such as a description of the conceptual journey of the scientist as they developed their ideas, make scientific ideas more engaging, memorable, and relatable for students (Britton et al. 1983, Graesser et al. 1994, Kim 1999). This section details essential scientific concepts, explains how the scientist developed their research question, and presents the experimental design and data collection methods, accompanied by images of the research process and study system.

After this step, students are given the scientists’ research question. Based on this question, students identify the scientist’s hypothesis, or the testable explanation for an observation (Strode 2015). Typically, hypotheses and predictions are embedded in the research background, requiring students to revisit that text and distinguish these two key elements of the scientific process (Strode 2015).

*Authentic dataset.* Next, students work with an authentic dataset derived directly from the featured research and explore data variability and trends. At this point of the activity, students shift from learning about the life and work of scientists to taking a more active role of engaging in the work of the scientist. When working with authentic datasets in DataVersify materials, students are able to actively participate in the process of constructing and applying knowledge, which has been shown to enhance student learning (Situated Learning Theory; Lave and Wenger 1991). Datasets support multiple learning outcomes, including exploring surprising results that do not support the hypothesis, applying quantitative reasoning skills in context of real-world phenomena, and understanding the nature of science (Kjelvik and Schultheis 2019; Schultheis and Kjelvik 2020; Strode et al. 2025).

Most datasets are provided in table form and curated from larger datasets to ease graphing and analysis using pencil and paper; they typically consist of one to three independent and one to three dependent variables with 10–50 data points each (Rosenberg et al. 2022; Schultheis et al. 2022), though vary in complexity (Kjelvik and Schultheis 2019). Students may need to perform basic statistical calculations before data visualization (e.g., mean or other measures of central tendency) and are often provided with measures of variability (e.g., standard deviation, standard error of the mean). Because of their authenticity, students grapple with datasets that may be missing values, have high levels of variability, or show surprising trends that do not follow predictions (Schultheis and Kjelvik 2020).

From the dataset, students are prompted to identify the independent and dependent variables relevant in the context of the provided scientific question and hypothesis (Angra and Gardner 2016, 2017; Abraham et al. 2025). Datasets might provide only two variables, or can be more complex where students must select the appropriate variables (Kjelvik and Schultheis. 2019). For many DataVersify activities, larger datasets from the research are made available to instructors, creating opportunities for students to explore additional variables and observations (Schultheis and Kjelvik 2020; Rosenberg et al. 2022). These larger datasets make possible advanced statistics for hypothesis testing, including confidence intervals, *t*-test, chi squared, regression, and analysis of variance (ANOVA) (see AP Biology statistics standards; College Board 2013).

*Data visualization.* After exploring the data in table form, students move on to create a visual representation of the dataset, typically in the form of a graph. Graphs are an essential tool within science, used to visualize data in order to communicate findings, explore patterns, and provide the evidence in support of a claim (Gardner et al. 2024). Thus, data visualization is an essential competency for students in biology (Angra and Gardner 2017; Clemmons et al. 2020). A key learning objective of DataVersify materials is to develop students’ metarepresentational competence, or students’ ability to create, interpret and evaluate the effectiveness of graphical representations of data (diSessa 2004).

DataVersify activities scaffold the challenging task of graph assembly (Gardner et al. 2024; Abraham et al. 2025) by providing three versions of the graphing task that can be used in sequence across activities as instructors help their students develop graphing abilities: *Graph type A* provides a completed graph made by the scientist, allowing students to focus on graph reflection and extracting meaning from the data visualization (Abraham et al. 2025). *Graph type B* provides axes and scales, but requires students to plot the data themselves. *Graph type C* offers blank graph paper; in this version, students must do additional graphing tasks such as choosing the appropriate graph type for the data provided and intended purpose (Webber et al. 2014; Abraham et al. 2025), placing the variables on appropriate axes, creating a scale, and creating legends when necessary for more than two variables (Angra and Gardner 2016, 2017), key competencies for graph assembly as described in the graph construction competency model for biology (Gardner et al. 2024, 2025; Abraham et al. 2025) and *Vision and Change* (Clemmons et al. 2020).

Often, DataVersify activities will include more data than is necessary to answer the scientific question and students must determine which independent and dependent variables need to be graphed in order to serve as support for their claims. Instructors can further adapt or scaffold the graphing task to align with students’ prior knowledge or learning goals.

Before moving on from their graphs, students are instructed to identify any changes, trends, or differences they see on their graphs, draw arrows pointing out what they see, and write one sentence describing how they interpret what they see. This instructional strategy, called Identify and Interpret (I^2^), was developed by BSCS Science Learning as a strategy to help students make sense of graphical information by breaking the task down into smaller parts (BSCS 2012a, 2012b). Within DataVersify, this prompt gives students the opportunity to observe their graphs and see what the data are telling them before moving on to answering the scientific question and drafting explanations.

*Scientific explanations.* After the graphing task, students are asked to construct scientific explanations to tell the story of the data. Within DataVersify, the explanation structure is scaffolded based on the claim-evidence-reasoning (CER) framework, which is widely adopted by educators and underlies the *Vision and Change* competencies, College Boards’s AP Biology standards, and NGSS’s practices for (1) constructing scientific explanations and (2) engaging in argument from evidence (College Board 2013; NGSS Lead States 2013; Clemmons et al. 2020). The CER framework, adapted from Toulmin’s (2003) model of argumentation, emphasizes that a scientific explanation must state a clear claim, support it with appropriate evidence, and use reasoning to justify why the evidence supports the claim (McNeill and Krajcik 2008). These prompts scaffold the explanation task and provide clear instructions for what to include within each component. As with the graphing task, instructors may choose to adapt and further scaffold this task, or remove the CER scaffold all together once students gain competency in this area.

After constructing their explanations, students return to the science research background to determine whether the data supported or did not support the scientist’s initial hypothesis. Because DataVersify activities are built from authentic investigations within biology, datasets represent an exploration of the unknown; in many cases, the data do not follow predictions and support the scientist’s hypothesis. Many datasets also have anomalous or unclear results, providing opportunities for complex student reasoning (Adams and Barth-Cohen 2024).

*Next steps.* In scientific practice, studies often reveal new avenues for exploration, provide opportunities to test reproducibility, and ask the same questions in new systems (Pelaez et al. 2022). At the end of a DataVersify activity, students ask new questions and generate their own ideas for subsequent steps that could extend the presented research. In this section, students are prompted to evaluate the strength of the evidence and describe what they think should be done next to build on the current work. This final section signals to students that research does not end at the conclusion of a study, but that each study in science builds upon previous work and inspires future questions (Schultheis and Kjelvik 2020).

*Teacher guides.* Each DataVersify activity has an associated teacher guide, which offers opportunities for customization, expansion, additional datasets for analysis, and connections to the primary scientific literature. Teacher guides often include additional details from the scientist about their study system and highlight areas where scientific themes can be extended and aligned with course content. Opportunities for class discussions or addressing possible student misconceptions are noted. Together, scaffolding and instructor guides allow instructors to customize DataVersify activities to work in their individual classroom context.

In some cases, instructors may choose to go beyond the DataVersify activity and have students conduct follow-up investigations. For example, when a study took place in a particular ecosystem, students may replicate the study’s methods in their local ecosystem and address similar questions. Or students can use larger datasets with additional variables to ask their own questions of the scientist’s system. In this way, DataVersify activities provide students with exposure to a scientist and their research, while preparing them to engage authentically in scientific inquiry by testing new hypotheses and addressing original questions.

## Efficacy research into DataVersify

Our collaborative team has used DataVersify activities as a model system to conduct research exploring how featuring scientist role models in the context of data literacy instruction influences undergraduate biology students’ perceptions of scientists (Schultheis et al. 2024; Costello et al. 2025a), which has in turn informed resource design and development. Specifically, we conducted a large-scale national experiment in introductory biology courses at over 40 institutions of higher education in the United States. To identify the effective elements of scientist role model profiles, we created different versions of DataVersify activities to serve as treatments manipulating the type and amount of information provided about the featured scientists, while keeping the scientific content constant across conditions. In a control treatment, we only mentioned the scientists’ names and pronouns; in a visual treatment, we additionally provided photographs of the featured scientists; in a humanizing treatment, we included the full previously described DataVersify scientist profile, including the Q&A (Schultheis et al. 2024; Costello et al. 2025a). Participating instructors implemented DataVersify activities from all three treatments in a randomized order across consecutive academic terms. Immediately following the DataVersify activities, students completed surveys assessing the extent to which they related to the featured scientists and their engagement with the DataVersify activities (Costello et al. 2025a).

We hypothesized the biographical elements in the humanizing treatment provided students with a more nuanced perspective on successful scientists, who may otherwise be considered unrelatable and their success unattainable (Costello et al. 2025a). Data from 3700+ students revealed that when students encounter data literacy materials enhanced with humanizing scientist information, they relate more to the featured scientists in a wide variety of ways, including relating to the scientists’ research interests, life experiences, and identities (Schultheis et al. 2024). The increase in relating to a scientist was especially strong for students who shared identities with the featured scientists and ultimately translated to higher student engagement (Costello et al. 2025a). When DataVersify activities included biographical information (i.e., their interests outside of science, the struggles they had to overcome in science) about the scientists who collected the data used in the activities, students put more effort into the activities, had more interest in the activities, and found the activities more relevant to their future (Costello et al. 2025a).

Our work has also addressed concerns that inclusive materials may elicit student resistance, defined by Seidel and Tanner (2013) as behaviors that reflect student frustration, detachment, or disengagement. As a post hoc analysis, we found that students expressed minimal resistance to these activities that highlighted scientists with counter-stereotypical identities (Costello et al. 2026). Together, our research calls for incorporating humanizing information about scientists featured in data literacy instruction and documents minimal outward resistance to doing so.

## Six evidence-based suggestions for how to implement activities featuring scientists with counter-stereotypical identities in biology

Results from the collaboration revealed several strategies that enhance the implementation of DataVersify activities and advance our fundamental understanding of what makes scientist role models effective in undergraduate biology. From our research, we have identified six lessons about featuring contemporary scientists in biology courses.

1. *Humanize scientist role models.* We found that students related more to featured scientists and engaged more in the DataVersify activities when they saw the full scientist profile along with the data literacy activity (i.e., the humanizing treatment: Costello et al. 2025a). DataVersify scientist profiles highlighted the obstacles the scientists overcame to get to where they are today, the identities they hold that are marginalized in STEM, and their hobbies outside of science. When humanizing information was shared, students related to scientists in a wide variety of ways: through their scientific interests, identities, hobbies, and interests outside of STEM (Schultheis et al. 2024). Simply providing photographs of scientists holding counter-stereotypical identities was not sufficient. As such, we encourage scientists to provide humanizing information about themselves, and for educational materials to contain more than a photo of the scientist alongside a description of their experiments and findings.
2. *Share a variety of contemporary scientists and their stories.* Students engage more in biology when a scientist featured in their course materials is relatable. Recent research showed textbooks over-represent scientists who possess stereotypical science identities and largely exclude humanizing information and photos of people (Wood et al. 2020; Richards et al. 2024). For example, Wood et al. (2020) found for every woman scientist that textbooks highlighted, they highlighted seven men scientists, and >96% of scientists featured in textbooks were white. Our research found that students who share identities with featured scientists related more to those scientists when humanizing information was included (Costello et al. 2025a). Therefore, the more types of scientist identities that are shared in a course, the more likely a student will be able to relate (Schultheis et al. 2024). Unfortunately, textbooks and other educational resources often fail to represent the range of people within the STEM field (Costello et al. 2025b), there are contemporary scientists doing all types of science all over the globe. Presenting scientists who reflect the identities and interests of your student population will foster engagement and learning.
3. *Focus on authenticity.* Using authentic data to teach data literacy skills can improve student critical thinking surrounding analyzing and interpreting data, as well as student ability to use evidence to back up scientific arguments (Kjelvik and Schultheis 2019; Schultheis et al. 2022). Beyond the focus on authentic datasets, several aspects of DataVersify activities are thoughtfully designed for authenticity, including the focus on the featured scientist’s story, access to their raw data, and the narrative description of the broader context of the featured research (Schultheis and Kjelvik 2015, 2020). Hypothesized elements of authentic scientific research that are highlighted by DataVersify include experiencing failure, iteration, grappling with messiness and complexity, scientific collaboration, engaging in scientific methodology, and autonomy, among others (Auchincloss et al. 2014; Rowland et al. 2016; Munn et al. 2017; Goodwin et al. 2021; Schultheis et al. 2022; Von der Mehden et al. 2023).
4. *Pair scientist stories with science content.* Curricular materials that highlight scientists with marginalized identities in biology courses and do not forefront their science may unintentionally delegitimize those scientists. As a result, students holding marginalized identities may feel tokenized in the biology classroom (as discussed in Costello et al. 2025b). Tokenism occurs in science classrooms when students perceive that scientists are referenced *because of* their difference from other scientists to serve as evidence that the dominant group does not discriminate (Zimmer 1988). Our DataVersify partnership aims to avoid tokenism by pairing authentic data collected by scientists with information about the real people behind the science.
5. *Implement in ways that work for your class.* We found that variation in instructor approaches to implementation did not impact the positive effect that the humanizing treatment had on undergraduate student outcomes (De Jesus et al. in prep). Specifically, we found that implementation style (homework, in-class discussion), incentivization (graded or ungraded), and format (online, in-person, and hybrid) did not explain variation in our data related to student engagement or relating to the scientist. These results show how benefits can occur across different instructor teaching decisions.
6. *Support education equity regardless of political landscapes.* Educational equity aims to provide all students with what they need to succeed in a learning environment (Ballen et al. 2025). Seeing oneself in science through the presence of relatable role models—or, conversely, recognizing one’s absence from the scientific enterprise—can significantly influence student retention and belonging in STEM (Seymour and Hunter 2019). Despite well-intentioned efforts, instructors have reported hesitations to implementing explicitly inclusive activities in their courses due, in part, to fears of student pushback or resistance (Beatty et al. 2023). In light of a challenging political climate, we searched for evidence of student resistance to the DataVersify activities, which are explicitly aimed at improving inclusivity in STEM by featuring scientists holding counter-stereotypical identities, through a post-hoc analysis of student survey responses. We found limited resistance to these activities (Costello et al. 2026). Only 16 student responses out of over 6400 total responses (or only 0.25% of student responses) were clearly resistant to the inclusive elements of the DataVersify activities. Our results counter instructor concerns and instead provide preliminary evidence that the overwhelming majority of students do not exhibit outward resistance to inclusive activities in their biology courses.

## Future of the DataVersify program

By merging quantitative biology and education research, the development of DataVersify as a framework has advanced our understanding of how to engage undergraduate biology students in data literacy. Our collaborative team continues to develop new DataVersify activities in collaboration with scientists, and pursues several active avenues of research.

*Obstacles.* Most students face obstacles throughout their academic career (Tracey et al. 2022), and tend to be aware of these obstacles (Prathibha et al. 2026). Individuals who have marginalized identities may face additional obstacles, resulting in higher rates of attrition (Hernandez et al. 2018). Ranging from academic challenges to social barriers, obstacles act as deterrents for such students when pursuing a career in STEM. However, students facing obstacles can achieve valued outcomes if they employ strategies to navigate these obstacles (Gin et al. 2018). While highlighting the success of scientists may portray a positive view of these individuals, it fails to realistically represent challenges students may face when pursuing a STEM career. The presentation of role models and their stories detailing how they encountered and overcame obstacles may motivate students to continue to pursue a STEM career (“If they can do it, I can too!”) (Lockwood 2006). The incorporation of scientist stories featuring obstacles is uniquely important because most exposure to contemporary scientists lacks this information, instead presenting less relatable and academically outstanding individuals. Future work will center the obstacles faced by scientists and how they overcame them. Examining how this information influences students’ perceptions of the challenges they might encounter in science will deepen our understanding of the impact of scientist narratives on undergraduate students. Ultimately, future research should investigate how stories about contemporary scientists can be used to support the academic pathways of undergraduate biology students.

*Success.* Stories of how scientist role models obtain and advance in their science careers can increase student motivation to pursue a science career too, but only when those paths are perceived as attainable (Gladstone and Cimpian 2021). Student’s perceptions of the various aspects of career success are central to understanding what achievement is and isn’t perceived as attainable. These perceptions of attainability may be dependent on underlying beliefs students have about who can and cannot achieve success in STEM (i.e., “I can’t do what they do”) (Limeri et al. 2023). By exploring whether there is a relationship between student’s perceptions of career success and their beliefs about who can succeed, we will be able to better understand why students may potentially react negatively when presented with successful role models, and develop interventions to overcome such reactions.

*Hobbies.* Students relate to many different aspects of the DataVersify scientist profiles, including information about their professional research interests (e.g., their research topic, their career trajectory) and their personal lives (e.g., their life experiences, identities, personality characteristics, and hobbies) (Schultheis et al. 2024). Hobbies may be a particularly effective way to help students relate to scientists and find connection and balance in their own future careers. Graduate students suffer high levels of stress and burnout up to six times greater than the general population (Chi et al. 2023), with poor work-life balance identified as a key driver (Jenkins et al. 2019; Zhou et al. 2020; Li et al. 2023). Our team will explore the impact of sharing scientists’ hobbies on burnout, specifically in biology graduate education. We developed scientist profiles highlighting scientists’ hobbies (see the Beyond the Bench; Lattin n.d.), and current efforts focus on implementing and evaluating these profiles in biology graduate education contexts, graduate courses and lab meetings. Ultimately, we aim to promote interest outside of science among biology graduate students to reduce burnout and alter the culture of productivity over wellbeing that is pervasive in academic science.

*Authenticity.* STEM education researchers have both hypothesized and demonstrated that engagement with authentic scientific practices and primary literature improves student scientific thinking, science identity, self-efficacy, motivation, engagement, and ultimately a student’s retention and graduation in their chosen STEM field (Hoskins et al. 2011; Eagan et al. 2013; Graham et al. 2013; Robnett et al. 2015). However, authentic science experiences are difficult to implement in the classroom, and rely on instructor and researcher perceptions of what authentic science in a classroom can look like. Our team will evaluate the elements of DataVersify activities that read as authentic to students, with the hope of enhancing and capitalizing on this authenticity to optimize future versions of the activities for improving an array of student outcomes. For example, we may observe that hearing about the stories of real scientists or manipulating and interpreting real data may enhance the authenticity of our curricular materials.

*Unexpected Results.* An original goal of the Data Nuggets program was to feature the often surprising and unexpected outcomes of authentic scientific research. As a result, Data Nuggets often feature data that contrast with the scientists’ hypotheses or predictions. For example, a Data Nuggets activity might feature alternative hypotheses, or the relationship between two variables might be null or opposite of what was predicted. Students may enter into their research careers believing that hypotheses generated by scientists are usually supported by their data, but preliminary data from a national survey found that the majority of biologists surveyed estimate that up to 40% of their research outcomes are unexpected (Graham et al. in prep). DataVersify and Data Nuggets activities ask students to engage with and interpret the featured scientist’s research outcomes, and whether they support the proposed hypothesis or expected pattern in the data. However, that’s where the story typically ends. New work by our team proposes to expand on this research outcomes angle by following up with featured scientists and asking them to discuss how the outcomes of the highlighted research impacted them and their career. Ultimately, we hope stories acknowledging and celebrating the varied outcomes of research will demonstrate to students that unexpected results are to be expected.

*Developing survey instruments supported by relevant validity evidence.* Although the benefits of materials featuring profiles of role models are well-documented, less is known about the specific features of scientist profiles that are most effective in maximizing their impact. This is in part due to inadequate instruments that measure impact. Future research in this area will focus on results from systematic reviews that have documented the most likely aspects that make role models effective, such as those documented in Gladstone and Cimpian (2021) or Morgenroth et al. (2015). For example, Gladstone and Cimpian proposed that three central features make an effective role model: the extent that students can relate to role models, their perceived competence, and the perceived attainability of their success. Currently there are few robust measures and none that we are aware of that focuses specifically on students’ perceptions of scientists. Therefore, future efforts to develop survey instruments supported by validity evidence will increase the rigor and value of research on role models in science.

## Conclusions

Since its inception in 2020, the DataVersify collaboration has collected evidence in support of sharing authentic, in-context research with students to support a range of cognitive and affective outcomes. Our activities are versatile and cover a wide array of topics in science, and our rigorous classroom-based research studies have allowed us to form evidence-based recommendations for their use in the undergraduate classroom. At the core, DataVersify activities build data literacy skills and promote student engagement in biology, while also highlighting counter-stereotypical scientists. Future studies featuring DataVersify materials will expand the variety of materials offered, and will explore the impacts of the decisions made when choosing which science stories to share with students. Focusing specifically on developing recommendations to promote data literacy will advance understanding of how authentic data experiences promote student learning. Our findings are broadly relevant, extending beyond this curricular resource to inform how instructors and mentors can achieve a variety of learning goals.

## Data Availability

See original publications for data associated with each study.
